# Evaluation of an Expert System for the Generation of Speech and Language Therapy Plans

**DOI:** 10.2196/medinform.5660

**Published:** 2016-07-01

**Authors:** Vladimir Robles-Bykbaev, Martín López-Nores, Jorge García-Duque, José J Pazos-Arias, Daysi Arévalo-Lucero

**Affiliations:** ^1^ Grupo de Investigación en Inteligencia Artificial y Tecnologías de Asistencia Universidad Politécnica Salesiana Cuenca Ecuador; ^2^ AtlantTIC Research Center for Information and Communication Technologies Department of Telematics Engineering University of Vigo Vigo Spain

**Keywords:** speech-language pathology, rehabilitation of speech and language disorders, decision support systems, clinical, expert systems

## Abstract

**Background:**

Speech and language pathologists (SLPs) deal with a wide spectrum of disorders, arising from many different conditions, that affect voice, speech, language, and swallowing capabilities in different ways. Therefore, the outcomes of Speech and Language Therapy (SLT) are highly dependent on the accurate, consistent, and complete design of personalized therapy plans. However, SLPs often have very limited time to work with their patients and to browse the large (and growing) catalogue of activities and specific exercises that can be put into therapy plans. As a consequence, many plans are suboptimal and fail to address the specific needs of each patient.

**Objective:**

We aimed to evaluate an expert system that automatically generates plans for speech and language therapy, containing semiannual activities in the five areas of hearing, oral structure and function, linguistic formulation, expressive language and articulation, and receptive language. The goal was to assess whether the expert system speeds up the SLPs’ work and leads to more accurate, consistent, and complete therapy plans for their patients.

**Methods:**

We examined the evaluation results of the SPELTA expert system in supporting the decision making of 4 SLPs treating children in three special education institutions in Ecuador. The expert system was first trained with data from 117 cases, including medical data; diagnosis for voice, speech, language and swallowing capabilities; and therapy plans created manually by the SLPs. It was then used to automatically generate new therapy plans for 13 new patients. The SLPs were finally asked to evaluate the accuracy, consistency, and completeness of those plans. A four-fold cross-validation experiment was also run on the original corpus of 117 cases in order to assess the significance of the results.

**Results:**

The evaluation showed that 87% of the outputs provided by the SPELTA expert system were considered valid therapy plans for the different areas. The SLPs rated the overall accuracy, consistency, and completeness of the proposed activities with 4.65, 4.6, and 4.6 points (to a maximum of 5), respectively. The ratings for the subplans generated for the areas of hearing, oral structure and function, and linguistic formulation were nearly perfect, whereas the subplans for expressive language and articulation and for receptive language failed to deal properly with some of the subject cases. Overall, the SLPs indicated that over 90% of the subplans generated automatically were “better than” or “as good as” what the SLPs would have created manually if given the average time they can devote to the task. The cross-validation experiment yielded very similar results.

**Conclusions:**

The results show that the SPELTA expert system provides valuable input for SLPs to design proper therapy plans for their patients, in a shorter time and considering a larger set of activities than proceeding manually. The algorithms worked well even in the presence of a sparse corpus, and the evidence suggests that the system will become more reliable as it is trained with more subjects.

## Introduction

Developing and maintaining proper communication skills is a mainstay for every individual to express needs, to learn, to be related with the environment and, in general, to have the opportunity to participate as an active member of society. According to the World Health Organization, individuals with communication difficulties are at a significant social disadvantage in both developing and developed countries [1]. This disadvantage often affects a person’s emotional and social life and can compromise educational and job opportunities, particularly in sectors where effective communication is critical, such as health care, education, local government, and justice.

Speech and language therapy (SLT) is an area of health care focused on the evaluation and treatment of a broad range of disorders, which can be roughly classified as affecting voice, speech, language, or swallowing capabilities. Disorders like selective mutism, dysarthria, aphasia, and dysphagia have a substantial impact on quality of life and human potential, whether they affect children who stutter as they struggle to speak up in class, lawyers or teachers with adult-onset voice disorders, or post-stroke individuals laboring to communicate verbally. Numerous studies about the incidence and prevalence of communication disorders in developed countries depict similar realities for Europe, Canada, Australia, and the United States [2-5]. Based on figures like the 7.5 million people in the United States who have voice disorders, the 3 million who stutter, and the 6-8 million who have been diagnosed with some form of language impairment, the American Speech-Language-Hearing Association estimates that 40 million Americans are affected by communication disorders, costing the nation US $154-184 billion annually [6]. It is estimated that more than 60 million people in the European Union are affected, with an estimated cost of €220-260 billion. As the population ages and survival odds improve for fragile infants and individuals who have sustained injury or acquired disease, the number of people with communication disorders will likely continue to increase [7].

Notwithstanding the societal and economic impact of communication disorders, SLT remains a largely overlooked area of health care. The latest World Report on Disability highlights that many countries suffer from lack of professionals, services, and structures to provide effective assessment, diagnosis, counseling, intervention, and treatment for people suffering from communication disorders [8]. In such conditions, speech and language pathologists (SLPs) have very limited time to work with their patients. This may mean that the diagnosis may fail to accurately identify the causes of the disorders, that the designed therapy plans may be suboptimal (eg, because the SLPs fail to keep in mind the whole set of activities they could apply), or that the treatment may be insufficient or not properly applied [7]. In this respect, Turnbull et al [9] found that only 19.2% of young people (from birth to 21 years old) who have communication disorders are actually receiving some form of specific care. Mackenzie et al [10] surveyed SLT provision for people with aphasia in the United Kingdom and found many areas reported low staffing levels and were thus unable to provide the recommended care or a comprehensive service. Code and Heron [11] also concluded that people with aphasia receive significantly less therapy than national recommendations suggest.

Over the last decade, many research efforts have separately shown evidence that the application of information and communication technology (ICT) has great potential to improve the quality and efficiency of SLT practice, as well as health outcomes and patients’ quality of life. There have been several approaches to automate diagnostic tests by means of audiovisual signal processing [12-15] and to automate the generation of therapy plans for specific disorders [16,17]. In this paper, we evaluate the support provided to SLPs by the SPELTA (SPEech and Language Therapy Assistant) expert system presented by Robles-Bykbaev et al [18], which aims to automatically generate therapy plans for SLT, containing semiannual activities and daily exercises for an unrestricted range of disorders affecting the five areas of hearing, oral structure and function, linguistic formulation, expressive language and articulation, and receptive language. The goal is to assess whether the expert system can speed up the SLPs’ work and lead to more accurate, consistent, and complete therapy plans for their patients.

## Methods

### SPELTA Expert System

**Figure 1:**
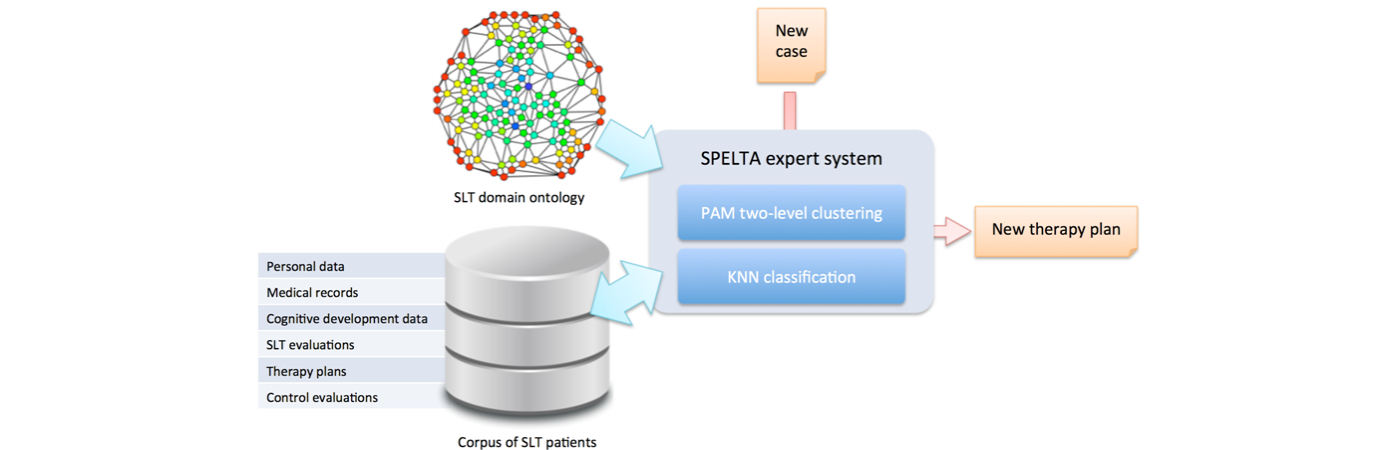
A block diagram of the SPELTA system.

The SPELTA expert system is one part of a set of ICT tools developed by Universidad Politécnica Salesiana (Ecuador) and Universidade de Vigo (Spain) to support SLT within an integrative environment for clinicians and students, pathologists, patients, relatives, and other potential users [19]. The environment is based on a formal knowledge model of the SLT domain and leans on OpenEHR solutions to support the storage and exchange of health-related data. As depicted in Figure 1, the SPELTA system is involved with the automatic generation of therapy plans for new subjects, based on two sources of information: (1) domain ontologies that interrelate the activities and the exercises with specific diseases, speech-language disorders, and skills, and (2) the corpus of patient profiles, containing the compendium of data, plans, and evaluations of previous patients.

Specifically, the profile of an SLT patient contains the following data:

- Personal data, including chronological age, gender, name, etc.
- A medical record specifying diagnosis, general medical conditions and related disorders (eg, cerebral palsy, hemiparesis, athetosis), as indicated by doctors.
- A record of cognitive development data, indicating cognitive age, gap in language development, expressive language age, and receptive language age (as estimated by SLPs).
- An SLT evaluation that looks at 102 parameters from the five SL areas:

1. Hearing—subjective evaluation of the auditory condition: reflex, localization of sound sources, and response to voice.

2. Oral structure & function—tongue, teeth, palate, lips, and maxillary mobility.

3. Linguistic formulation—phonation and breathing condition.

4. Expressive language and articulation—vocal development, social communication, semantics (content)-vocabulary and concepts, structure (form)-morphology and syntax, and integrative thinking skills; pronunciation of phonemes, sentences, polysyllabic words, and vowel phonemes.

5. Receptive language—attention, semantics (context)-vocabulary and concepts, structure (form)-morphology and syntax, and integration skills.

- A therapy plan, containing five subplans with lists of semiannual activities and daily exercises for each one of the SL areas. One example of activity could be “perform blow exercises to increase the blowing force.” Two specific exercises related to this activity could be “blow confetti 10 times during 2 seconds” or “inflate one balloon in no more than 6 exhalations.”
- Control evaluations with the results of successive therapy sessions.

**Figure 2:**
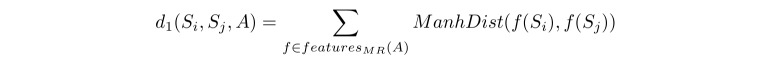
Metric used to determine the distance between two subjects in a specific SL area, according to their profile.

Internally, the SPELTA system relies on an implementation of the Partition Around Medoids algorithm to generate clusters of patient profiles with two levels of granularity [20]. The generation of a new therapy plan is dealt with as a classification problem, looking for the most similar cases in each one of the five SL areas according to the K-Nearest Neighbors criterion [21]. First-level clusters represent groups of patients who may have similar speech-language skills and limitations, but possibly arising from (or linked to) different medical conditions. To create these groups, we use the distance metrics of Figure 2, where *S*_i_ and *S*_j_ refer to two different subjects, *A* is one of the SL areas, *f* goes over the set of features from the medical records relevant for that area (*features*_MR_*(A)*), and ManhDist denotes the mean-Manhattan binary distance [18].

**Figure 3:**
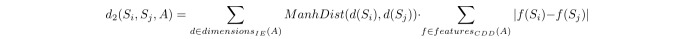
Metric used to determine the distance between two subjects within a specific first-level cluster.

In the second level, the subjects are clustered according to the fine-grained evaluation of the record of cognitive development data and the initial SLT evaluation. For example, within a first-level cluster that includes the cases of children with Down syndrome and phonological disorders, we need to differentiate subjects who commit additions (ie, adding extra sounds in some words, eg, “balue” for “blue”) from subjects who commit substitutions (ie, one or more sounds are substituted for others, eg, “bagon” for “wagon”). In this case, we use the distance metrics of Figure 3. The first summation measures the mean-Manhattan binary distance of the initial SLT evaluations of two subjects, considering only the dimensions relevant to the speech-language area in question, *dimensions*_IE_*(A)*. The second summation provides a scale factor derived from the absolute differences of cognitive age, gap in language development, expressive language age, and receptive language age (the features of cognitive development data) [18].

**Figure 4:**
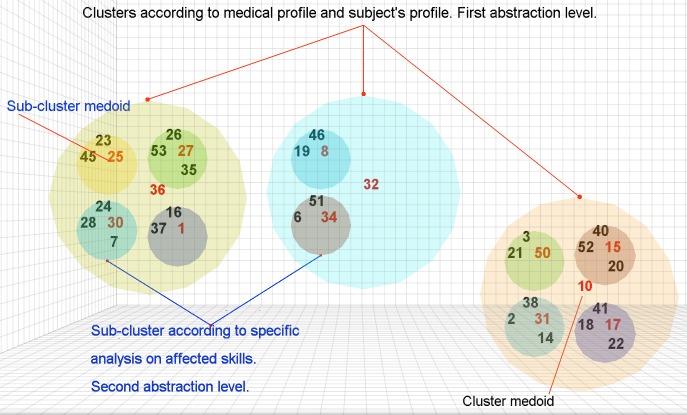
The clustering approach of the SPELTA system. This structure is used for each speech-language area.

Figure 4 depicts an example of the cluster structure generated by SPELTA for each of the SL areas we consider. Each one of the first-level and second-level clusters has one of the subject cases designated as a medoid, rather than a fictitious case computed by averaging. This facilitates the classification of new cases, identifying the closest subjects in each one of the SL areas.

The plans provided by the SPELTA system are presented to SLPs through visual interfaces, so that they can validate it as a whole or modify certain parts, as they deem necessary. To facilitate the task, the interfaces show which cases were found to be closest in each one of the SL areas. If several subjects were found to be equally distant to the new one in some of the areas, then it is possible to browse the superset of activities, the intersections, and the disjunctions. As an example, Table 1 shows the activities of one master plan generated by the SPELTA system, with the third column indicating the most similar subjects in each area and the features that make them similar to the new case. The profile description is as follows: age 15 years, 8 months; medical diagnosis of athetoid cerebral palsy (ICD-10-CM code G80.3); speech and language diagnosis of mixed receptive-expressive language disorder (ICD-10-CM code F80.1); receptive language age of 4 years; expressive language age of 2 years, 8 months; and a language developmental age of 3 years, 4 months.

**Table 1:** The activities of a sample therapy plan provided by the SPELTA system (Case 52).

Area	Activities	Source subplans
Hearing	Perform exercises to sounds identification.	Case 37: a patient with a similar receptive language age (4 years, 6 months) and a 100% coincidence in the evaluation of hearing (cochleo-palpebral reflex, startle response, turns head to sound source, identifying sound objects, sound source localization without visual stimulus).
	Discriminate sounds of nature, body, and animals.	
	Perform phonemes discrimination exercises.	
Oral structure & function	Perform segmental relaxation massages.	Case 18: a patient with an 84% coincidence in the oral peripheral mechanism (same tongue size, same speed in tongue movements, present tongue protrusion, voluntary and involuntary swallowing are present, is able to chew hard and soft food, sialorrhea is not present).
	Perform slow and fast tongue movements.	
	Perform exercises with lips (retraction and protrusion).	
	Achieve sound productions using the oropharynx structure.	
	Perform active and passive exercises using tongue, lips, and jaw.	
Linguistic formulation	Work in the automatic respiration process (inspirations and expirations), and work with blow exercises to increase the blowing force.	Case 22: a patient with a 70% coincidence in linguistic formulation (same respiratory frequency, same thorax symmetry, diaphragmatic breathing).
	Respiration exercises associated to vowels and simple phonemes (/pa/, /da/, /fo/).	Case 3: a patient with a 70% coincidence in linguistic formulation (diaphragmatic breathing, no nasal obstruction, same exhalation period).
Expressive language & articulation	Construct sentences from a given word.	Case 22: a patient with a similar expressive language age (1 year, 7 months), similar diagnosis for the medical examination (cerebral palsy and mixed receptive-expressive language disorder) and a 100% coincidence in the speech-language evaluation.
	Sort out the words of a sentence.	
	Work in grammatical structure.	
	Develop the spontaneous conversation	
	Perform activities that use twisters and rhymes.	
	Work with the personal articulation exercise book.	
Receptive language	Work with sequences and puzzles of 4 elements.	Case 37: a patient with a similar receptive language age (4 years, 6 months), similar diagnosis for the medical examination (cerebral palsy and mixed receptive-expressive language disorder) and a 90% coincidence in the speech-language evaluation (the only difference relates to the use of place prepositions like “under,” “over,” etc).
	Learn semantic categories	
	Identify objects according to their utility.	
	Identify daily activities.	
	Learn temporal notions (day and night, before and after).	
	Identify similar/distinct objects according to their utility.	

### Study Participants and Data Preparation

For the study presented in this paper, the SPELTA expert system was deployed, along with the accompanying tools, in three special education institutions for children in Ecuador: Instituto de Parálisis Cerebral del Azuay (Institute of Cerebral Palsy of Azuay), Fundación “General Dávalos” (“General Dávalos” Foundation), and CEDEI School. Over the course of 2 years (from September 2012 to September 2014), a team of 4 SLPs progressively created a corpus of 117 children profiles, including the corresponding number of therapy plans created manually by themselves and subsequent control evaluations. Some relevant data from the corpus are included in Multimedia Appendix 1. The most common conditions were those of cerebral palsy with/without accompanying dysarthria, dyslalia, epilepsy or dysphasia (n=22), Down syndrome with/without dysarthria or dysphasia (n=19), intellectual disability with/without dysarthria or dysphasia (n=10), autistic disorders (n=9), and fetal alcohol syndrome (n=5). These are the disorders with greatest prevalence in the Ecuadorian province of Azuay.

The corpus is admittedly small and sparse, implying that certain conditions may occur only a few times and many combinations are not included. However, that sparsity is a representative feature of the SLT area because the range of disabilities and communication disorders is so broad that even if two cases have the same medical diagnosis and similar patient profiles, they can require largely different therapy strategies or the support of different assistive technologies. The SPELTA expert system was precisely designed bearing this problem in mind.

**Figure 5:**
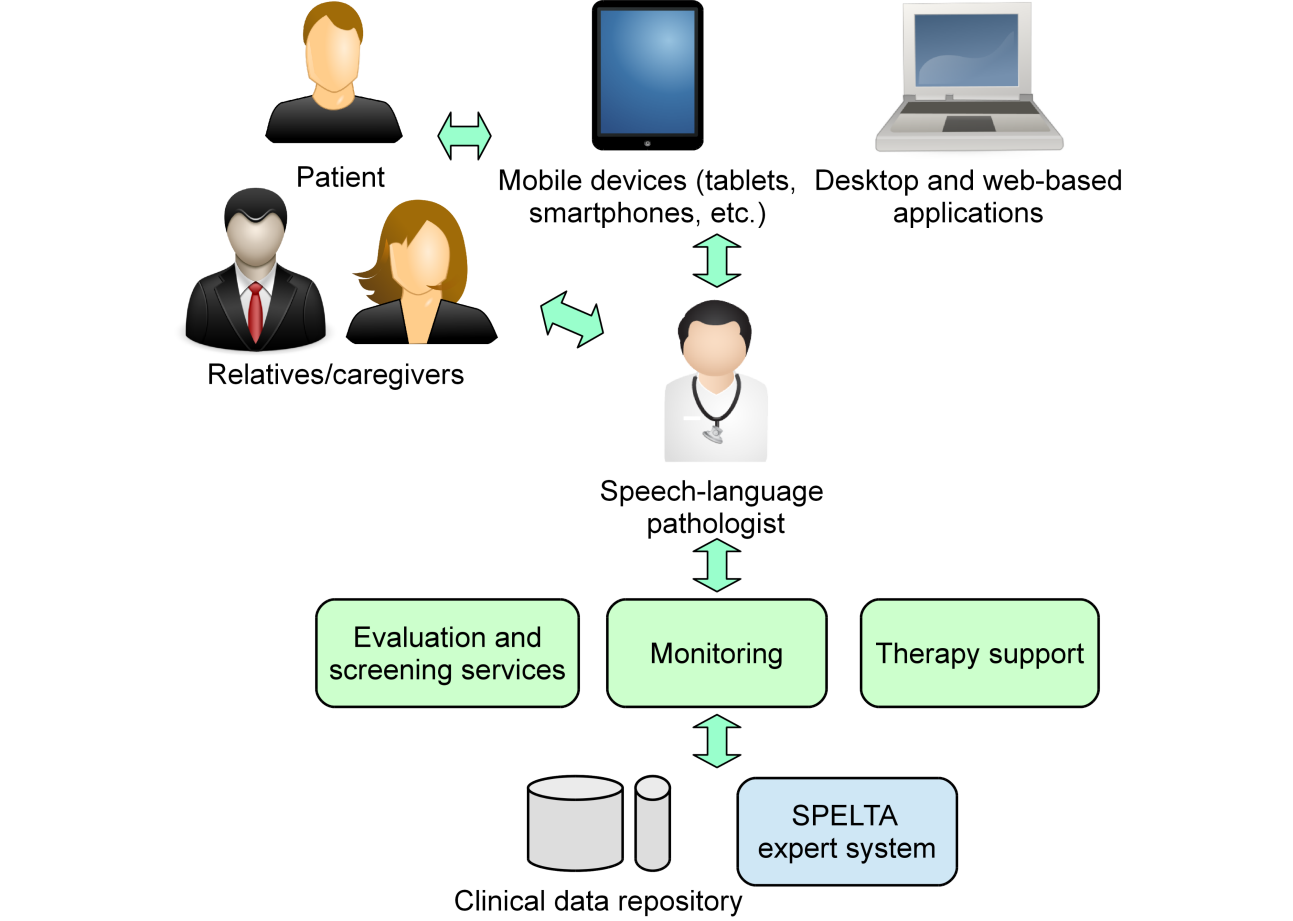
Diagram of the interfaces and services provided by the SPELTA system.

**Figure 6:**
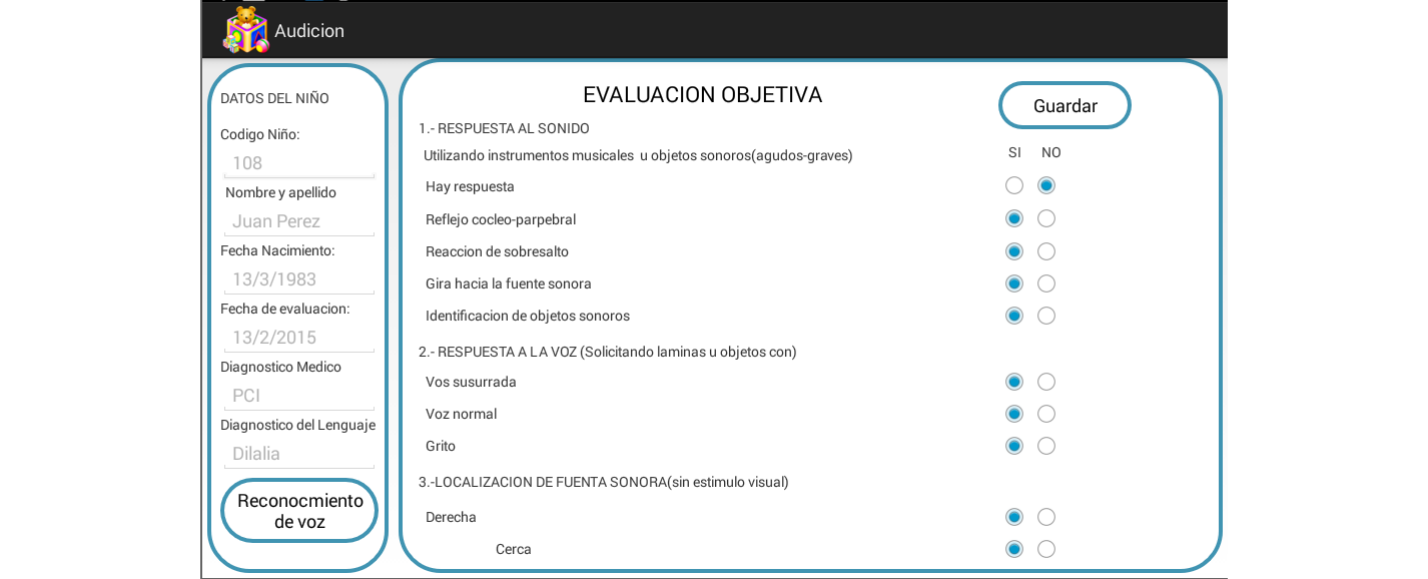
Screen capture of the hearing test that can be applied with mobile devices.

**Figure 7:**
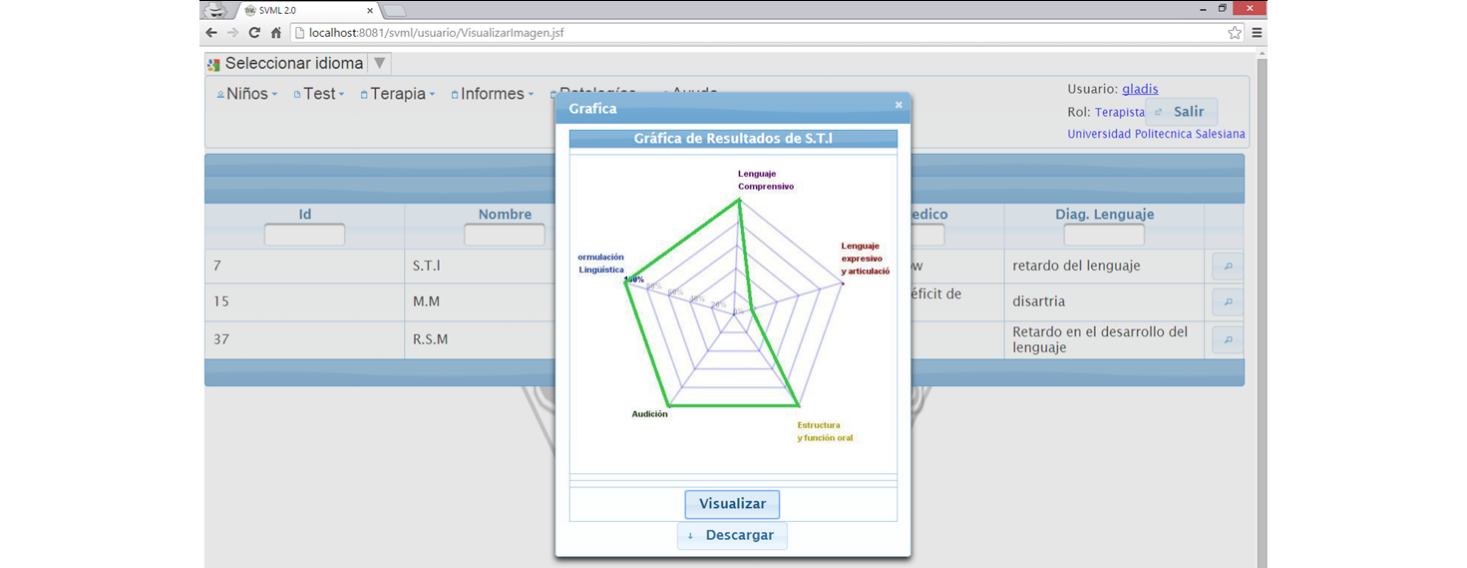
Webpage showing the results of patients' skills in the five SLT areas.

**Figure 8:**
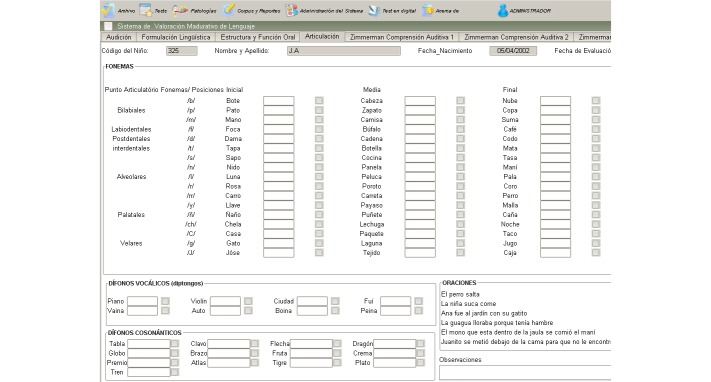
Screen capture of the articulation test on a desktop application.

The collaborating SLPs used the interfaces and services provided by SPELTA to perform an initial screening of each patient, followed by a personalized evaluation of the 102 SL parameters, and finally, the manual design of a proper therapy plan. As shown in Figure 5, the tools were available on mobile devices as well as desktop computers (see Figures 6-8). The patients could use smartphones or tablets to engage in interactive exercises to evaluate some speech-language skills or to receive memory, motor, hearing, and visual stimulation. The mobile apps proved very useful for SLPs to annotate data about patients who suffer from disabilities that affect their motor skills (eg, cerebral palsy, hemiparesis, hemiplegia) because they allow working in a comfortable space for the patient at work or home. In turn, the desktop apps were most useful with patients in a consulting room or in the rehabilitation centers, and to provide remote assistance.

### Evaluation Method

Having trained its algorithms on the corpus of 117 cases and the corresponding plans, the first stage of the evaluation of the SPELTA expert system involved the generation of therapy plans for the cases of 13 new children (see Multimedia Appendix 2). The SLPs discussed whether each one of the automatically generated plans was convenient or not, considering the following criteria.

#### Accuracy

The exercises and activities selected by SPELTA must be adequate to support the development and rehabilitation of one or more skills related to speech and language. For example, if a patient needs to improve speech production, it is necessary that they have proper breathing conditions and adequate control of their lips and tongue. The accuracy criterion refers to whether the exercises and activities within a plan match the skills that should be improved in the patient.

#### Consistency

Each patient’s profile has different characteristics, such as medical diagnosis, developmental language age, chronological age, etc. The consistency criterion is used to analyze whether a plan contains exercises and activities that can be carried out in a proper way with each patient, bearing in mind their capacity to understand the requests, the affected skills, the developmental gap, etc. For example, cases 23 and 32 (see Multimedia Appendix 1) represent two patients suffering from Down syndrome who had similar developmental language ages (a difference of only 1 month). However, case 23 presented a developmental gap of 2 years and 1 month, whereas case 32 had a 5-year gap. The consistency criterion provides for dealing with these two cases with different activities and exercises, even though the profiles are similar in terms of medical diagnosis and developmental age.

#### Completeness

In order to have an effective rehabilitation plan, it is necessary to have an adequate number of exercises and activities (not too many or too few). In this line, the completeness criterion is used to determine whether the number and complexity of exercises is adequate for a specific patient. For example, the plan in Table 1 (generated by the SPELTA system) contains the following activities for the hearing area: perform exercises to sounds identification, discriminate sounds of nature, body and animals, and perform phonemes discrimination exercises. The collaborating SLPs confirmed that those guidelines are appropriate to help developing the skills that allow patients to identify phonemes, to construct words and short sentences, and to develop auditory memory over a period of 6 months. Similarly, the number of knowledge areas related to communication is properly delimited for a patient who has a receptive language age of 4 years.

**Figure 9:**
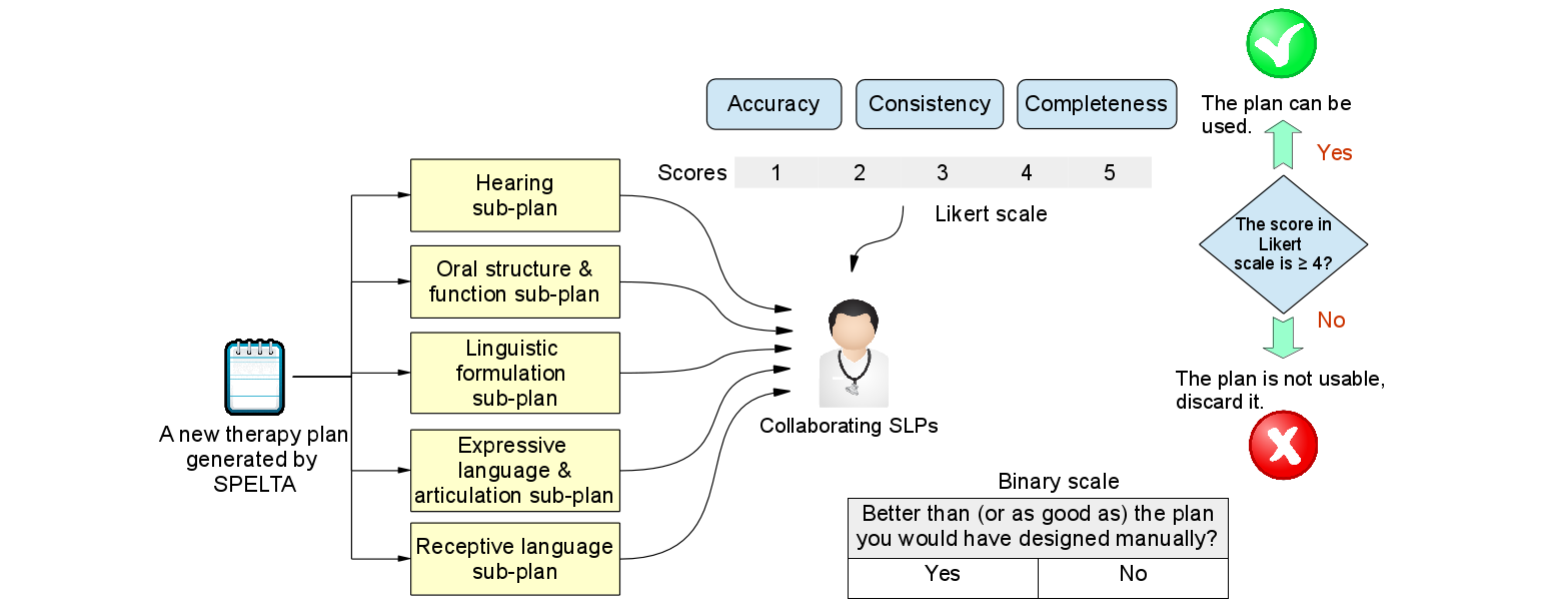
The evaluation process followed to assess the plans provided by the SPELTA expert system.

As shown in Figure 9, these criteria were assessed separately for the five subplans of each new plan generated by the SPELTA system, that is, looking at the activities and exercises assigned to each of the five SLT areas. The collaborating SLPs would rate accuracy, consistency, and completeness of each subplan on a 5-point Likert scale, and only the ones that achieved average scores ˃4 were considered valid and were to be used during the therapy process. Additionally, each SLP would provide a binary response to whether each subplan was “better than” or “as good as” the subplan they would have created manually if given the average time that they could devote to the task.

In order to get further evidence about the statistical significance of the results, we made the experiment to evaluate the SPELTA expert system using a 4-fold cross-validation approach. Specifically, we partitioned the original corpus into 4 sets of 29, 29, 29, and 30 cases, and each cross-validation round consisted of asking the system to provide therapy plans for the cases of each subset, after training it with the cases of the 3 others. The SLPs would discuss whether each one of the automatically generated plans was convenient or not, as above.

## Results

### Generation of Therapy Plans for New Cases

**Figure 10:**
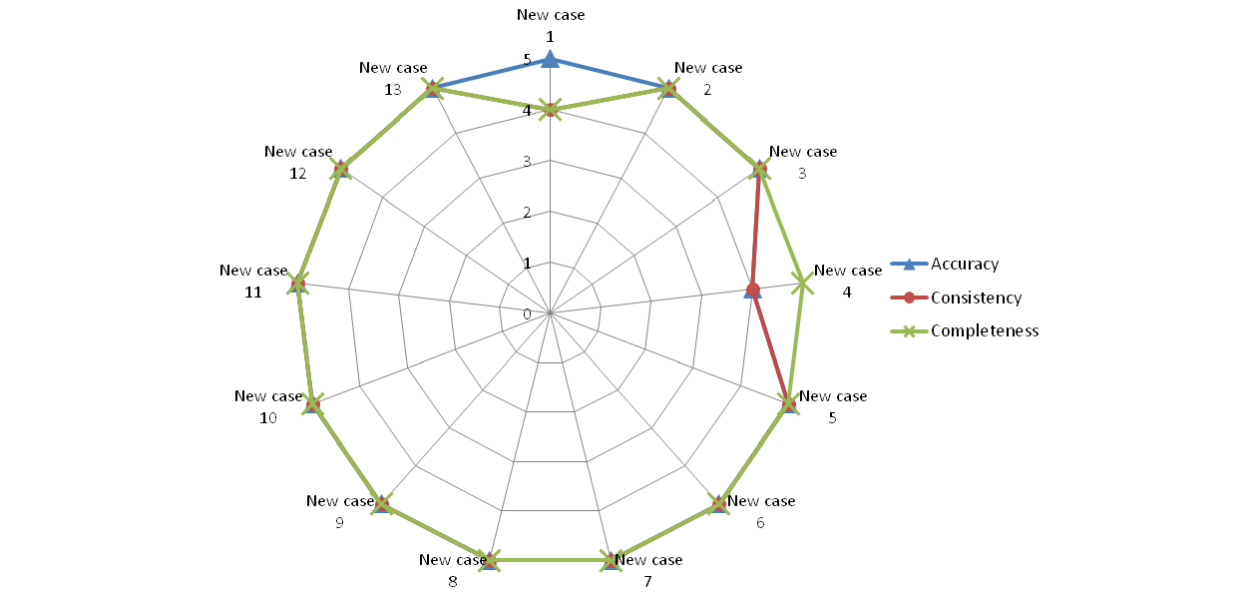
Results achieved by the expert system in the area of hearing.

**Figure 11:**
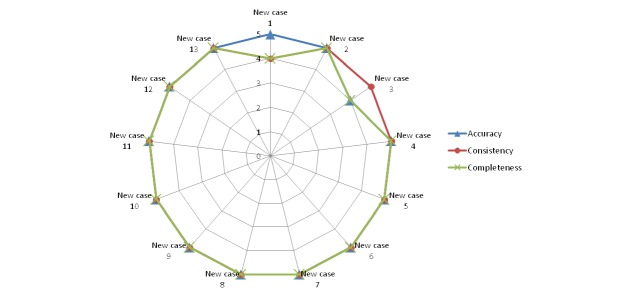
Results achieved by the expert system in the area of oral structure and function.

**Figure 12:**
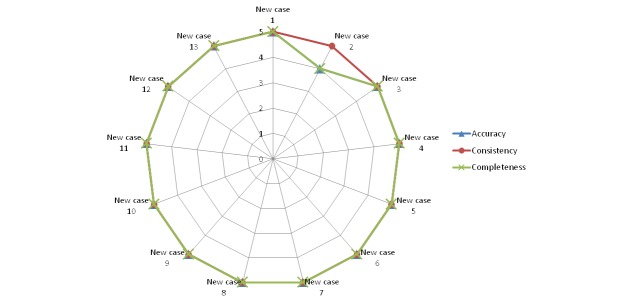
Results achieved by the expert system in the area of linguistic formulation.

**Figure 13:**
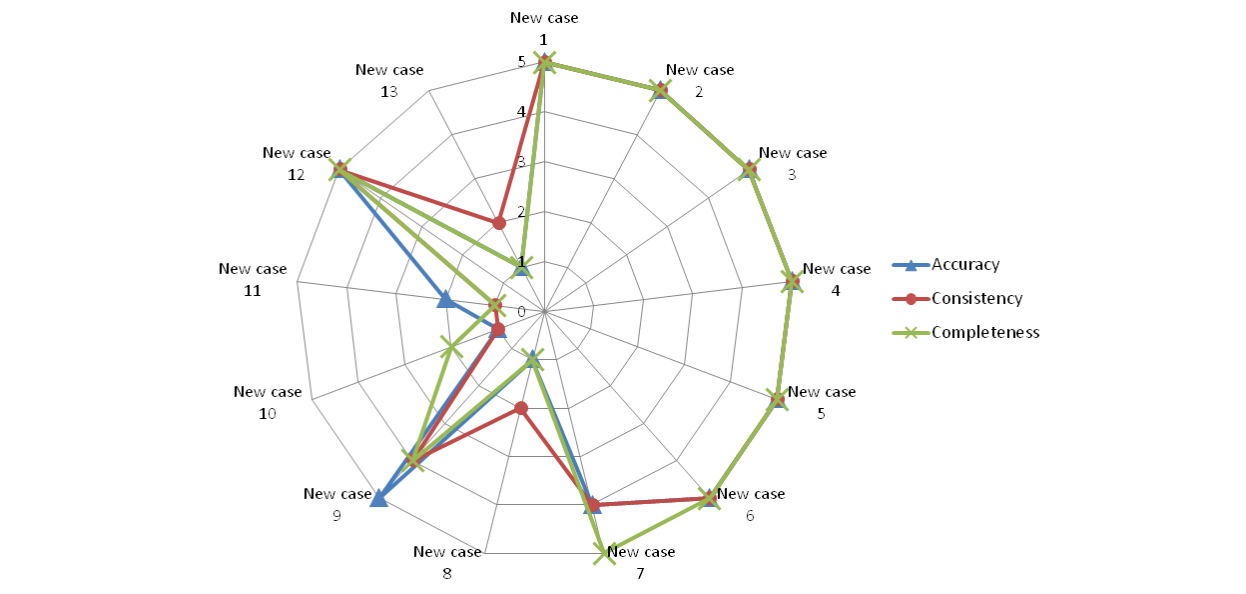
Results achieved by the expert system in the area of expressive language and articulation.

**Figure 14:**
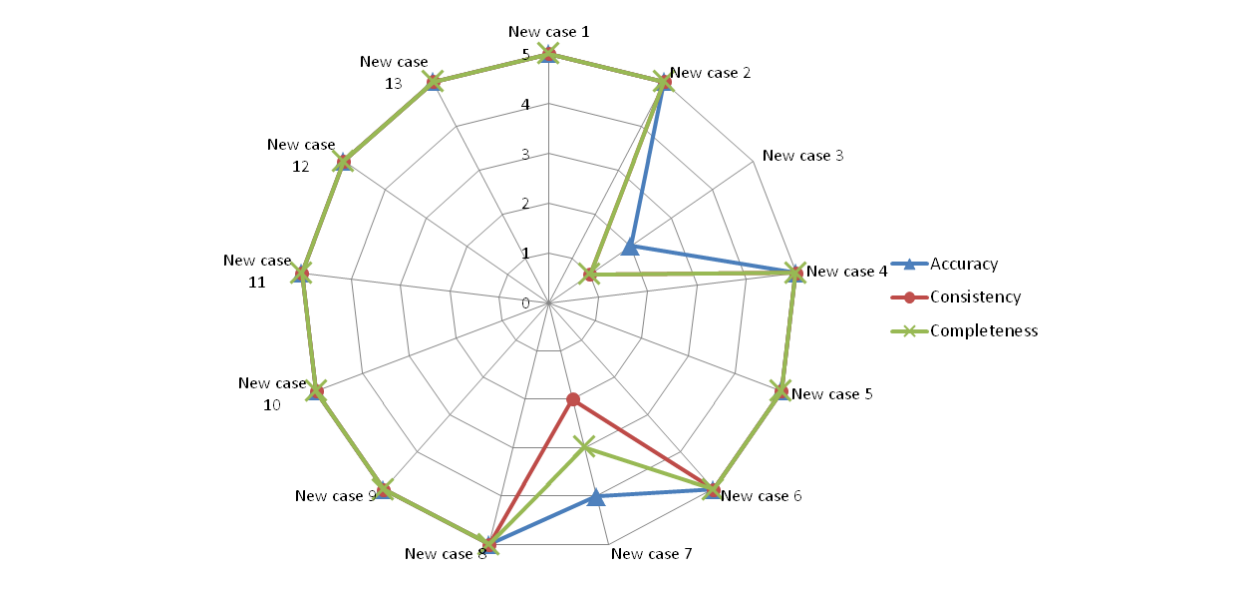
Results achieved by the expert system in the area of receptive language.

Figures 10-14 show the average values obtained on the Likert scale for each of the subplans provided by the SPELTA system when given the input of the 13 new cases: Figure 10 shows the results in the SLT area of hearing, Figure 11 shows oral structure and function, Figure 12 shows linguistic formulation, Figure 13 shows expressive language and articulation, and Figure 14 shows receptive language. The three criteria (accuracy, consistency, and completeness) are represented with different line colors. We can make the following observations per area.

#### Hearing

The 13 subplans generated for this area were considered usable by the SLPs according to the Likert scale. Indeed, only the subplans assembled for new cases 1 and 4 obtained scores of 4 in some of the criteria; all other ratings were 5. For case 1 (a patient with Down syndrome), the SLPs found that it was possible to make some small improvements in the consistency and completeness of the subplan, for which they added one activity to reinforce auditory memory through exercises related to the execution/understanding of simple orders. For case 4 (a patient with mild intellectual disability), in turn, the SLPs determined that the subplan provided by SPELTA was complete for the selected activities, but these did not fully address all the necessary skills in a fully consistent manner for the patient. They changed two activities for less complex ones and added one activity to stimulate the localization of sound sources.

#### Oral Structure and Function

Again, the 13 subplans generated for this area were considered usable, and only the ones generated for new cases 1 and 3 obtained lower than perfect ratings. Regarding case 1, the SLPs considered the subplan largely usable and were looking at fine-grained details due to their abundant experience in treating Down syndrome. For case 3 (a patient with spastic cerebral palsy and dysphasia), the subplan was found to be fully consistent with the patient’s needs, but some of the selected activities were not the best for the case, and the routines missed some exercises the SLPs deemed important. Driven by the most similar cases available in the training corpus, the SPELTA system selected a few exercises that were more suitable for someone with a slightly greater developmental age (around 4 years).

#### Linguistic Formulation

In this area, all subplans provided by SPELTA were considered usable, and only the one designed for new case 2 (a patient with spastic hemiparesis and dysphasia) got scores of 4 for accuracy and completeness. The SLPs found it necessary to include exercises to complement oral motor rehabilitation and to develop some mainstays (eg, lips control, tongue control) that would provide support in more complex process (eg, getting correct positioning of the phono-articulatory organs for speech production).

#### Expressive Language and Articulation

This is the area where the expert system showed poorest performance, since it failed to generate usable subplans for new cases 8, 10, 11, and 13. The SLPs found that some of the selected activities would not serve to train the affected skills (inaccuracy), whereas some of the exercises were too complex for the ages and developmental gaps of those patients (inconsistency), and the overall planning of the therapy sessions was not balanced, lacking attention to important traits (incompleteness). The analysis of the cases revealed that the training corpus was too sparse to address their specifics according to the outcomes of the evaluation of the 102 SL parameters. In the absence of very specific training, for example, SPELTA produced largely similar subplans for the new cases 8 and 10, reusing activities and exercises from previous cases that were found to be similar. However, even though both subjects were affected by athetoid cerebral palsy, they differed in that subject 8 would not understand some orders and exercises, whereas subject 10 would not be able to perform some of the selected exercises due to uncontrolled movements of limbs and trunk.

#### Receptive Language

In this area, the system could not generate a correct subplan only for the cases 3 and 7. The subplan generated for case 7 (a patient affected by cerebral palsy) would have been better suited to someone with greater developmental age, whereas the one generated for case 3 (a patient with spastic cerebral palsy and dysphasia) failed to pay proper attention to the large developmental gap.

The average values of accuracy, consistency, and completeness attained in the five SL areas and globally are shown in Table 2. The validity of the subplans generated automatically and of the therapy plans as a whole (discarding any plan that contained an invalid subplan) are given in Table 3.

Finally, Table 4 summarizes the replies to the question of whether the subplans provided by SPELTA were “better than” or “as good as” the plans that the SLPs would have created manually.

**Table 2:** Average values of accuracy, consistency, and completeness.

	Hearing	Oral structure & function	Linguistic formulation	Expressive language & articulation	Receptive language	Overall
Accuracy	4.92	4.92	4.92	3.77	4.69	4.65
Consistency	4.85	4.92	5	3.77	4.46	4.60
Completeness	4.92	4.85	4.92	3.77	4.54	4.60

**Table 3:** Validity of the subplans generated for each area, and the plans as a whole.

Subplans	%
Hearing	100
Oral structure & function	100
Linguistic formulation	100
Expressive language & articulation	69
Receptive language	85
Overall plans for the five areas	54

**Table 4:** Percentage of positive replies to whether the expert system provided an output comparable to that of a human SLP.

Subplans	%
Hearing	100
Oral structure & function	85
Linguistic formulation	92
Expressive language & articulation	62
Receptive language	85
Overall plans for the five areas	92

### Cross-Validation on a Partition of the Corpus

Tables 5,6, and 7 show the average values obtained on the Likert scale in the four rounds of cross-validation with a partition of the original corpus of 117 cases. In turn, Tables 8 and 9 contain data about the validity of the therapy plans and subplans provided by the system, and the replies to the question of whether the subplans were “better than” or “as good as” the plans that the SLPs would have created manually.

**Table 5:** Average values of accuracy, consistency, and completeness for the areas of hearing and of oral structure and function in the rounds of cross-validation.

K	Hearing			Oral structure & function		
	Accuracy	Consistency	Completeness	Accuracy	Consistency	Completeness
1	4.8	4.74	4.91	4.94	4.97	4.75
2	4.93	4.87	4.9	4.95	4.85	4.87
3	4.84	4.8	4.83	4.82	4.91	4.83
4	4.9	4.72	4.85	4.92	4.83	4.77
Average	4.87	4.78	4.87	4.91	4.89	4.81

**Table 6:** Average values of accuracy, consistency, and completeness for the areas of linguistic formulation, and of expressive language and articulation in the rounds of cross-validation.

K	Linguistic formulation			Expressive language & articulation		
	Accuracy	Consistency	Completeness	Accuracy	Consistency	Completeness
1	4.84	4.94	4.72	2.93	2.93	2.68
2	4.89	4.98	4.85	2.78	2.91	2.97
3	4.8	4.89	4.91	3.57	3.02	3.31
4	4.9	4.82	4.9	3.14	2.98	3.16
Average	4.86	4.91	4.85	3.11	2.96	3.03

**Table 7:** Average values of accuracy, consistency, and completeness for receptive language and overall scores in the rounds of cross-validation.

K	Receptive language			Overall		
	Accuracy	Consistency	Completeness	Accuracy	Consistency	Completeness
1	4.57	4.01	4.28	4.42	4.32	4.27
2	4.67	4.41	4.51	4.44	4.40	4.42
3	4.66	4.41	4.55	4.54	4.41	4.49
4	4.34	4.65	4.28	4.44	4.40	4.39
Average	4.56	4.37	4.41	4.46	4.38	4.39

**Table 8:** Validity of the subplans generated for each area, and the plans as a whole in the rounds of cross-validation.

Subplans	1	2	3	4	Average
Hearing	97%	93%	97%	100%	97%
Oral structure & function	93%	93%	100%	100%	97%
Linguistic formulation	97%	100%	93%	93%	96%
Expressive language & articulation	79%	72%	72%	77%	75%
Receptive language	76%	76%	79%	80%	78%
Overall plans for the five areas	48%	45%	52%	50%	49%

**Table 9:** Percentage of positive replies to whether the expert system provided an output comparable to that of a human SLP in the rounds of cross-validation.

Subplans	1	2	3	4	Average
Hearing	97%	90%	93%	97%	94%
Oral structure & function	83%	79%	90%	83%	84%
Linguistic formulation	93%	93%	90%	93%	92%
Expressive language & articulation	55%	55%	59%	57%	57%
Receptive language	83%	79%	79%	87%	82%
Overall plans for the five areas	90%	93%	90%	90%	91%

## Discussion

### Principal Findings

The results obtained in this experiment of generating therapy plans for new subject cases are encouraging about the potential use of the SPELTA expert system in SLT practice. The ratings achieved in terms of accuracy, consistency, and completeness show that the system succeeds in the task of automatically creating new therapy plans out of the knowledge contained in its corpus and in the catalogues of activities and exercises. The subplans generated for the different SL areas were most often considered valid and directly usable, whereas the evaluation of the overall plans was hindered only by the relatively poor performance in the area of expressive language and articulation. Careful analysis of the results in that area suggests that it is necessary to refine some aspects of the reasoning mechanisms of the expert system, even though a more extensive corpus of cases would have also helped to achieve better ratings.

Overall, the SLPs found that the plans provided by SPELTA are, most often, as good as the ones they would have created themselves in their normal work routines (not given sufficient time to work optimally). Thus, the system is a useful tool that can achieve significant savings of valuable and scarce human resources. In order to substantiate the time savings, the SLPs informally measured that the identification and supervision of semi-annual activities to put in a new therapy plan went from an average of 30 minutes down to 5 minutes; the selection of multimedia resources for specific exercises and sessions went from 40 to 6 minutes; and the generation of reports was automated to the point of reducing 24 minutes to 3.

The percentage of positive judgments (92%; Table 4) is much higher than the percentage of plans that contained valid subplans for all five SL areas (54%; Table 3), showing that the SLPs still considered most of the subplans useful and valuable. Accordingly, the SLPs always took the output of SPELTA as a starting point to produce the final therapy plans to use with new patients. Furthermore, they praised the fact that the expert system helped them consider a larger set of activities and exercises than if they had proceeded manually.

The four rounds of the cross-validation experiment yielded similar results, but the fact that the training sets were smaller (87, 88, 88, and 88 cases against 117) had an impact on the quality of the therapy plans, going down from 4.65 accuracy to 4.46, from 4.60 consistency to 4.38, and from 4.60 completeness to 4.39. Still, 49% of the plans were valid straightaway, and 91% were received positively by the SLPs. The greatest impact of working with a more reduced knowledge base was seen in the area of expressive language and articulation, which is in line with the previous observation that a larger corpus will be beneficial.

### Comparison With Prior Work

Decision Support Systems (DSS) are becoming increasingly used in the realm of speech and language therapy, with plenty of technical solutions in place to address the specific challenges of the many different disorders. Some DSS depend entirely on input provided by humans, while others rely on signal processing techniques to achieve a level of automation. Thus, on the one hand, Martín Ruiz et al [22] evaluated a Web-based DSS to monitor children’s neurodevelopment via the early detection of language delays at a nursery school, relying on input provided by the educators and on a set of over 100 rules to generate alerts in case deviations from the expected developmental milestones. On the other hand, Schipor et al [12] presented a model for automatic assessment of pronunciation quality for children, using Hidden Markov Models (HMM) and implementing a correlation measure to measure the level of intelligibility of utterances. Similarly, Saz et al [13] had used HMM in combination with a subword-based pronunciation verification method. Utianski et al [14] developed an application able to record speech samples and make calculations to assess the integrity of speech production (vowel space area, assessment of an individual’s pathology fingerprint, and identification of parameters of the intelligibility disorder). For a final sample, Caballero-Morales and Trujillo-Romero [15] improved the recognition rates for dysarthric patients by integrating multiple pronunciation patterns using genetic algorithms.

All of the aforementioned works focused on providing aids for SLT diagnosis tasks. The idea of aiding in the design of speech and language therapy plans—as we aim to do with the SPELTA system—has fewer precedents in the literature. The closest reference can be found in the work of Schipor et al [16], who developed a system based on fuzzy logic to plan sessions for the treatment of dyslalia, taking input from social, cognitive, and affective parameters, and providing output about types of exercises, frequency, and duration. Later, Yeh et al [17] presented an approach based on neural networks to classify a wide range of SLT problems in order to help design occupational therapy plans, which may include some help to improve communication skills.

### Limitations

We believe our study has two main limitations. First, while the results do not show much variability (ratings of 5 were most numerous by far), the SPELTA system needs to be evaluated on a larger set of subject cases. Presumably, the system algorithms will behave more reliably in the presence of a larger corpus, since the sparsity of the corpus we used in our study was one of the reasons for the poor performance in the area of expressive language and articulation.

Second, and probably more important, it would be interesting to experiment with more SLPs from more institutions and other situations than in Ecuador. The 4 SLPs participating in our study had been trained by the same books in the same school, which raises the possibility that there might be some bias in the judgment of the therapy plans presented to them. In the quest for greater evidence, we are actively seeking agreements to test our tools with universities, foundations, and professional associations from other Spanish-speaking countries.

### Conclusions

Our study shows that the SPELTA expert system provides valuable input for SLPs to design proper therapy plans for their patients, in a shorter time and considering a larger set of activities than proceeding manually. The system achieves nearly perfect performance in the areas of hearing, oral structure and function, and linguistic formulation, and also decent performance in receptive language. The poorer results in the area of expressive language and articulation have served to identify opportunities for technical improvements, in order to deal properly with new combinations of medical conditions and SL disorders, not properly captured in the corpus. Having a more extensive corpus would obviously help, but in the meantime before a database with thousands of cases becomes available, we are doing research on whether it would be good to adjust internal parameters of the current reasoning system of SPELTA, to define new metrics to compare cases and profiles, and to supplement the internal logic with radically different machine learning artifacts such as the cortical learning algorithm [23].

For future work, we propose a study of two new artificial intelligence techniques supporting the generation of therapy plans. First, we want to use template-based generation methods with weak supervisions [24], defining a structure based on different levels of granularity in which it will be possible to incorporate common strategies, activities, and resources according to some specific traits and needs derived from the patient’s profile. Second, we are interested in deep belief networks and recurrent neural networks [25], which may be able to extract the subtlest patterns from the complex data and interrelations of the SLT area.

## Appendix

Multimedia Appendix 1 Summary of patient profiles.

Multimedia Appendix 2 Summary of profiles for patients randomly selected to the expert system.

## Abbreviations

DSS: Decision Support Systems
HMM: Hidden Markov Models
ICT: information and communication technology
SL: Speech and Language
SLP: Speech and Language Pathologist
SLT: Speech and Language Therapy
SPELTA: SPEech and Language Therapy Assistant
