# Supplementary material for: Evaluation of an Expert System for the Generation of Speech and Language Therapy Plans
Source: JMIR Med Inform. 2016 Jul 1;4(3):e23. doi: 10.2196/medinform.5660 (PMC4947192; doi:10.2196/medinform.5660)
Supplement: Multimedia Appendix 1 [file medinform_v4i3e23_app1.pdf]

Multimedia Appendix 1

The following table summarizes the profiles of the patients contained in our study, including ID (case number), chronological age, medical diagnosis, ICD-10-CM (International Classification of Diseases, version 10, Clinical Modification), developmental language age, and developmental gap (difference between chronological and language ages).

Case	Age	Medical diagnosis	ICD-10-CM Code	Developmental language age	Developmental gap
1	6y 7m	Spastic hemiparesis (left side)	G81	3y 9m	2y 10m
2	10y 10m	Down syndrome	Q90	4y 2m	6y 8m
3	10y 4m	Moderate intellectual disability	F71	6y 5m	3y 11m
4	5y	Down syndrome	Q90	1y 1m	4y 11m
5	8y 5m	Autistic disorder, attention-deficit hyperactivity disorder	F84.0; F90.1	4y 8m	3y 9m
6	10y 5m	Moderate intellectual disability; dysarthria	F71; R47.1	6y 1m	4y 4m
7	12y 8m	Down syndrome	Q90	4y 6m	8y 2m
8	10y 9m	Dysarthria	R47.1	5y 8m	5y 1m
9	10y 10m	Cerebral palsy; dysarthria	G80; R47.1	5y 9m	5y 1m
10	11y 3m	Cerebral palsy; dyslalia	G80; F80.0	1y	10y 2m
11	14y 5m	Autistic disorder	F84.0	5y 1m	9y 4m
12	17y 11m	Down syndrome	Q90	5y 8m	12y 3m
13	4y 11m	Autistic disorder	F84.0	3y 7m	1y 4m
14	10y 10m	Moderate intellectual disability	F71	4y 5m	6y 5m
15	7y 7m	Hyperactivity disorder; dysarthria	F90.1; R47.1	6y	1y 1m
16	6y	Moderate intellectual	F71; F80.1	1y 6m	4y 6m

		disability; dysphasia			
17	3y 4m	Dyslalia	F80.0	2y 9m	7m
18	5y 1m	Dyslalia	F80.0	3y 7m	1y 6m
19	5y 1m	Visual impairment	H54.3	3y 11m	1y 2m
20	33y 8m	Cerebral palsy; dysarthria	G80; R47.1	2y 6m	31y 2m
21	12y 3m	Cerebral palsy; dysarthria	G80; R47.1	1y 7m	10y 8m
22	5y 9m	Down syndrome	Q90	1y 3m	4y 6m
23	3y 4m	Down syndrome	Q90	1y 3m	2y 1m
24	5y 5m	Hyperactivity disorder; mild intellectual disability	F90.1; F70	3y 3m	2y 2m
25	11y 4m	Cerebral palsy; epilepsy	G80; G40.8	3y 7m	7y 9m
26	26y 1m	Down syndrome; dysarthria	Q90; R47.1	3y 1m	23y
27	10y 8m	Dysphasia	R47.0	4y 3m	6y 5m
28	14y 8m	Down syndrome; dysphasia	Q90; R47.0	4y 3m	10y 5m
29	10y 9m	Cerebral palsy; dysarthria	G80; R47.1	4y 1m	6y 8m
30	5y	Cerebral palsy; dysarthria	G80; R47.1	4y 6m	4m
31	10y 11m	Autistic disorder	F84.0	1y 7m	9y 4m
32	6y 4m	Down syndrome	Q90	1y 4m	5y
33	17y 9m	Cerebral palsy	G80	1y 3m	16y 6m
34	8y 8m	Cerebral palsy	G80	1y 9m	6y 11m
35	10y 9m	Polyneuropathy congenital; dysphonia	A50.43; R49.0	1y	9y 9m
36	18y 8m	Cerebral palsy	G80	3y 8m	15y
37	10y 1m	Cerebral palsy	G80	3y 6m	6y 7m
38	10y 4m	Cerebral palsy	G80	4y 1m	6y 3m

39	10y 6m	Specific developmental disorder of motor function	F82	1y 8m	8y 10m
40	11y 6m	Severe intellectual disability	F72	2y 3m	9y 3m
41	9y 4m	Spastic hemiparesis	G81	4y 5m	5y 11m
42	3y 2m	Dyslalia	F80.0	2y 10m	1y 4m
43	3y 8m	Dyslalia	F80.0	3y 7m	1m
44	5y 3m	Other developmental disorders of speech and language	F80.8	3y 4m	2y 11m
45	4y 7m	Other developmental disorders of speech and language	F80.8	3y 6m	1y 1m
46	5y 8m	Dyslalia	F80.0	5y 2m	6m
47	6y 1m	Other developmental disorders of speech and language	F80.8	5y 0m	1y 1m
48	4y 10m	Hyperactivity disorder	F90.1	3y 1m	1y 9m
49	3y 5m	Speech and language development delay due hearing loss	F80.4	1y 6m	2y 11m
50	5y 1m	Down syndrome	Q90	1y 6m	4y 7m
51	3y 8m	Dyslalia	F80.0	2y 10m	1y 10m
52	3y 6m	Epilepsy	G40.8	2y 1m	1y 5m
53	4y 7m	Dyslalia	F80.0	4y 9m	10m
54	6y 9m	Dyslalia	F80.0	3y 5m	3y 4m
55	3y 10m	Dyslalia	F80.0	1y 9m	2y 1m
56	2y 9m	Other developmental disorders of speech and language	F80.8	2y 4m	5m
57	3y 1m	Other developmental disorders of speech and language	F80.8	2y 1m	1y
58	1y 9m	Other developmental disorders of speech and	F80.8	1y 3m	6m

		language			
59	2y 5m	Other developmental disorders of speech and language	F80.8	1y 6m	1y 11m
60	2y 7m	Other developmental disorders of speech and language	F80.8	1y 6m	1y 1m
61	4y 7m	Expressive language disorder	F80.1	2y 11m	2y 8m
62	3y 11m	Other developmental disorders of speech and language	F80.8	3y 1m	10m
63	4y 8m	Dyslalia	F80.0	2y 6m	2y 2m
64	3y 11m	Dyslalia	F80.0	2y 7m	1y 4m
65	5y 10m	Dyslalia	F80.0	5y 0m	10m
66	4y 5m	Speech and language development delay due hearing loss	F80.4	1y 0m	3y 5m
67	4y 3m	Other developmental disorders of speech and language	F80.8	2y 7m	2y 8m
68	3y 2m	Other developmental disorders of speech and language	F80.8	2y 4m	1y 10m
69	4y 3m	Other developmental disorders of speech and language	F80.8	3y 5m	1y 10m
70	3y 3m	Other developmental disorders of speech and language	F80.8	1y 9m	2y 6m
71	5y 1m	Other developmental disorders of speech and language	F80.8	4y 7m	1y 6m
72	2y 11m	Other developmental disorders of speech and language	F80.8	1y 10m	1y 1m
73	5y 7m	Other developmental disorders of speech and	F80.8	4y 9m	1y 10m

		language			
74	4y	Other developmental disorders of speech and language	F80.8	1y	3y
75	5y 7m	Other developmental disorders of speech and language	F80.8	5y	7m
76	2y	Other developmental disorders of speech and language	F80.8	1y 6m	1y 6m
77	4y	Dyslalia	F80.0	3y 7m	1y 5m
78	5y 1m	Dyslalia	F80.0	4y 6m	1y 7m
79	4y 9m	Dyslalia	F80.0	4y 4m	5m
80	5y 2m	Dyslalia	F80.0	4y 9m	1y 5m
81	3y 2m	Dyslalia	F80.0	3y 7m	7m
82	4y 3m	Other developmental disorders of speech and language	F80.8	3y 7m	1y 8m
83	9y 8m	Down syndrome	Q90	2y 10m	7y 10m
84	10y 9m	Cerebral palsy	G80	3y 3m	7y 6m
85	8y 3m	Down syndrome	Q90	1y 11m	7y 4m
86	10y 9m	Down syndrome	Q90	3y 0m	7y 9m
87	7y	Autistic disorder	F84.0	2y 1m	5y 11m
88	9y 2m	Fetal alcohol syndrome; other developmental disorders of speech and language	Q86.0; F80.0	2y 10m	7y 4m
89	2y 7m	Other developmental disorders of speech and language	F80.8	1y 10m	1y 9m
90	3y 10m	Dyslalia	F80.0	3y 3m	7m
91	4y 11m	Dyslalia	F80.0	4y 3m	8m
92	10y 8m	Physical retardation due malnutrition	E45	5y	5y 8m
93	9y 3m	Cerebral palsy	G80	1y 3m	8y

94	6y 7m	Autistic disorder	F84.0	1y 3m	5y 4m
95	9y	Fetal alcohol syndrome; other developmental disorders of speech and language	Q86.0; F80.0	3y 3m	6y 9m
96	9y	Autistic disorder	F84.0	1y 9m	8y 3m
97	11y 9m	Down syndrome	Q90	3y 7m	8y 2m
98	6y 11m	Epilepsy and recurrent seizures	G40	2y	4y 11m
99	12y 2m	Fetal alcohol syndrome; other developmental disorders of speech and language	Q86.0; F80.0	3y	9y 2m
100	9y 4m	Physical retardation due malnutrition	E45	3y 11m	6y 5m
101	10y 2m	Autistic disorder	F84.0	5y 3m	5y 11m
102	11y 8m	Autistic disorder	F84.0	2y 4m	9y 4m
103	11y 2m	Fetal alcohol syndrome; other developmental disorders of speech and language	Q86.0; F80.0	2y 4m	9y 10m
104	7y 11m	Physical retardation due malnutrition	E45	3y 3m	4y 8m
105	12y 4m	Dandy-Walker syndrome	Q03.1	5y 6m	7y 10m
106	8y 11m	Down syndrome	Q90	1y 9m	7y 2m
107	8y 8m	Down syndrome	Q90	2y 1m	6y 7m
108	7y	Apert's syndrome	Q87.0	2y 4m	5y 8m
109	6y 8m	Physical retardation due malnutrition	E45	2y 4m	4y 4m
110	8y 1m	Fetal alcohol syndrome; Other developmental disorders of speech and language	Q86.0; F80.0	4y 9m	4y 4m
111	5y 11m	Down syndrome	Q90	2y 4m	3y 7m
112	6y 1m	Other developmental disorders of speech and	F80.8	2y 4m	4y 9m

		language			
113	12y 5m	Cerebral palsy	G80	3y 10m	9y 7m
114	6y 9m	Other developmental disorders of speech and language	F80.8	2y	4y 9m
115	11y 5m	Cerebral palsy	G80	1y 9m	10y 8m
116	9y 1m	Down syndrome	Q90	1y 8m	8y 5m
117	6y 7m	Severe intellectual disability	F72	1y	5y 7m
