# Supplementary material for: Evaluation of an Expert System for the Generation of Speech and Language Therapy Plans
Source: JMIR Med Inform. 2016 Jul 1;4(3):e23. doi: 10.2196/medinform.5660 (PMC4947192; doi:10.2196/medinform.5660)
Supplement: Multimedia Appendix 2 [file medinform_v4i3e23_app2.pdf]

Multimedia Appendix 2

The following table summarizes the profiles of the patients randomly selected to test the expert system.

Case	Age	Medical diagnosis	ICD-10-CM Code	Developmental language age	Developmental gap
1	7y 2m	Down syndrome	Q90	2y 4m	4y 10m
2	11y 6m	Spastic hemiparesis; dysphasia	G81; F80.1	2y 4m	9y 2m
3	16y 4m	Cerebral palsy (spastic); dysphasia	G80; F80.1	2y 1m	14y 3m
4	11y 11m	Mild intellectual disability	F70	3y 2m	8y 9m
5	12y 3m	Mild intellectual disability	F70	2y 8m	9y 7m
6	13y 8m	Epilepsy	G40.8	3y 4m	10y 4m
7	6y 3m	Cerebral palsy	G80	3y 3m	3y
8	15y 9m	Athetoid cerebral palsy	G80.3	3y 2m	12y 7m
9	8y 3m	Cerebral palsy (spastic)	G80	4y 9m	3y 6m
10	8y 1m	Athetoid cerebral palsy	G80.3	5y 3m	2y 10m
11	12y 10m	Moderate intellectual disability	F71	5y 3m	7y 7m
12	13y 11m	Spastic hemiparesis; dysphasia	G81; F80.1	5y 7m	8y 4m
13	4y 10m	Visual impairment	H54.3	1y 1m	3y 8m
